# DNA Microarray and Bioinformatic Analysis Reveals the Potential of Whale Oil in Enhancing Hair Growth in a C57BL/6 Mice Dorsal Skin Model

**DOI:** 10.3390/genes15050627

**Published:** 2024-05-15

**Authors:** Junko Shibato, Fumiko Takenoya, Ai Kimura, Michio Yamashita, Satoshi Hirako, Randeep Rakwal, Seiji Shioda

**Affiliations:** 1 Department of Functional Morphology, Shonan University of Medical Sciences, 16-48 Kamishinano, Totsuka-ku, Yokohama-shi 244-0806, Kanagawa, Japan; rjunko@nifty.com; 2 Department of Sport Sciences, School of Pharmacy and Pharmaceutical Sciences, Hoshi University, 2-4-41 Ebara, Shinagawa-ku 142-8501, Tokyo, Japan; kuki@hoshi.ac.jp (F.T.); francfranc.fragola@gmail.com (A.K.); d1902@hoshi.ac.jp (M.Y.); 3 Department of Health and Nutrition, University of Human Arts and Sciences, Saitama-shi 339-8539, Saitama, Japan; satoshi_hirako@human.ac.jp; 4 Institute of Health and Sport Sciences (TAIIKU), Global Sport Innovation, University of Tsukuba, 1-1-1 Tennodai, Tsukuba-shi 305-8574, Ibaraki, Japan; plantproteomics@gmail.com

**Keywords:** DNA microarray, oil, minoxidil, hair growth, immunostaining, animal model

## Abstract

Much research has been conducted to determine how hair regeneration is regulated, as this could provide therapeutic, cosmetic, and even psychological interventions for hair loss. The current study focused on the hair growth effect and effective utilization of fatty oil obtained from Bryde’s whales through a high-throughput DNA microarray approach in conjunction with immunohistochemical observations. The research also examined the mechanisms and factors involved in hair growth. In an experiment using female C57BL/6J mice, the vehicle control group (VC: propylene glycol: ethanol: water), the positive control group (MXD: 3% minoxidil), and the experimental group (WO: 20% whale oil) were topically applied to the dorsal skin of the mouse. The results showed that 3% MXD and 20% WO were more effective than VC in promoting hair growth, especially 20% WO. Furthermore, in hematoxylin and eosin-stained dorsal skin tissue, an increase in the number of hair follicles and subcutaneous tissue thickness was observed with 20% WO. Whole-genome transcriptome analysis also confirmed increases for 20% WO in filaggrin (*Flg*), a gene related to skin barrier function; fibroblast growth factor 21 (*Fgf21*), which is involved in hair follicle development; and cysteine-rich secretory protein 1 (*Crisp1*), a candidate gene for alopecia areata. Furthermore, the results of KEGG pathway analysis indicated that 20% WO may have lower stress and inflammatory responses than 3% MXD. Therefore, WO is expected to be a safe hair growth agent.

## 1. Introduction

In addition to protecting the head from external stimuli such as temperature, ultraviolet rays, and bruising, hair also excretes toxic substances such as heavy metals. Beautiful hair also has a significant impact on the visual effect of significantly changing one’s appearance and on the psychological effect of increasing one’s self-confidence. The demand for hair-care products is growing, especially with the recovery of economic activity due to the easing of behavioral restrictions caused by the COVID-19 pandemic, and the increasing number of elderly people and the growing problem of hair loss are expected to contribute significantly to future growth in the hair-care market. Currently, two drugs, minoxidil (MXD) and finasteride, are approved by the U.S. Food and Drug Administration (FDA) for the treatment of hair loss. Although the details of MXD’s mechanism of action are unknown, it is known to prolong the duration of the hair growth phase by inducing vasodilation of the scalp [1,2,3,4]. Finasteride has been demonstrated to prevent male pattern baldness by inhibiting 5α-reductase activity, which affects the metabolism of male hormones [5]. However, side effects such as scalp problems, including rashes and itching have been reported with MXD, and liver dysfunction, erectile dysfunction, and rashes have been reported with finasteride [6,7]. Thus, natural ingredients and organic formulations are in high demand in the hair-care product market.

Studies have shown that fish oil, which is rich in n-3 polyunsaturated fatty acids such as eicosapentaenoic acid (EPA), docosapentaenoic acid (DPA), and docosahexaenoic acid (DHA), has high antioxidant capacity, reduces systemic inflammation, and is generally known to prevent atherosclerosis [8,9]. Intake of foods with such antioxidant capacity and circulation-promoting effects is known to contribute to various health-promoting effects, and it has been reported that intake of foods with high antioxidant capacity promotes hair growth. Human hair is mostly made of amino acids, but fatty acids are also constituents of hair, and a decrease in fatty acids is known to lead to a decrease in water retention in the hair texture and further to a decrease in hair luster. In addition, a recent systematic review of hair growth and supplements [10] listed about a dozen dietary supplements that promote hair growth, including omega-3 and -6, which contain antioxidants. In addition, omega-3 fatty acids have blood flow-promoting properties, which may promote blood flow to the hair matrix cells in the hair follicles, which are important for hair growth and development in particular. There have also been reports of omega-3 and its effectiveness in promoting hair growth [11].

Hair growth repeats cycles of growth, regression, resting, and hair loss. The number of dermal papilla cells (DPCs) fluctuates throughout the hair cycle, and depletion or reduction in the number of DPCs results in the inability to re-enter the growth phase of the hair cycle [12,13]. In addition, since the increase in growth-promoting factors secreted by DPC is considered to be a very important parameter for evaluating hair-growth-promoting properties, it is necessary to maintain the function of DPC in order to maintain the normal hair cycle. Animal and vegetable oils rich in polyunsaturated fatty acids have been reported to improve scalp dermatitis and alopecia [14]. Arachidonic acid, which is structurally similar to omega-6 polyunsaturated fatty acids, promotes hair growth by prolonging the growth phase of the hair cycle [15] and also promotes dermal papilla cell viability and promotes FGF-7. It is known that hair formation is promoted by enhancing the expression of hair-growth-promoting factors [16]. Immortalized Human Follicle Dermal Papilla Cells (HFDPCs) are used in the development and evaluation of hair growth products because DPCs play an important role in hair formation and regulating hair cycle events. Therefore, we conducted an in vitro test to determine whether WO has the effect of proliferating HFDPCs.

Recent studies have confirmed that cold-pressed whale oil (WO) has high antioxidant and anti-inflammatory effects [17,18]. The high stearic acid content of the WO makes it suitable as a cosmetic material since the stearic acid in WO is a component that helps improve the barrier function and is used in conditioners and treatments. Furthermore, WO contains a large amount of DPA and EPA, which could be applied to scalp care and hair growth products. Experiments were conducted to confirm the hair-growth-promoting effects of WO, aiming to make effective use of the parts that are normally discarded and to utilize WO as a functional ingredient.

## 2. Materials and Methods

### 2.1. Sample Preparation and Whale Oil (WO)

Bryde’s whale (*Balaenoptera brydei*) skin was extracted with heated steam at 200 °C for 20 min and frozen. The WO was provided by Kyodo Senpaku Co. Ltd. (Tokyo, Japan).

### 2.2. MTT Assay

Immortalized Human Follicle Dermal Papilla Cells (HFDPCs; Cat. No.T0500, Applied Biological Materials Inc. (Abm) Richmond, BC, Canada) were seeded in 96-well culture plates at 3 × 10^4^ cells/100 μL/well and pre-cultured for 24 h at 37 °C and 5% CO_2_. Cultures were grown in DMEM (Dulbecco’s Modified Eagle’s Medium “Nissui” (2), Nissui Pharmaceutical) medium with 10% FBS (fetal bovine serum) and 1% antibiotic (Sigma A5955, St. Louis, MO, USA) at a ratio of 1%. After preincubation, the medium was replaced with new medium, and the WO, docosapentaenoic acid (DPA), and eicosapentaenoic acid (EPA) were adjusted to a concentration of 0.0–5.0% with D/E solution (DMSO:Ethanol = 1:1), and as a positive control minoxidil (MXD), which is used in hair growth products, was added at 5–30 μM concentration to D/E solution. Then, 24–120 h of incubation at 37 °C and 5% CO_2_ was followed by incubation with MTT (3-[4,5-]dimethylthiazol-2-2) from the Cell Growth Kit I (Roche Life Science, Indianapolis, IN, USA). After incubation at 37 °C and 5% CO_2_ for 4 h, 100 μL/well of solubilizing solution (0.01 M HCl, 10% SDS) was added and incubated at 37 °C and 5% CO_2_ for 24 to 120 h. After that, 100 μL/well of solubilizing solution (0.01 M HCl, 10% SDS) was added and incubated for 17 h at 37 °C, 5% CO_2_. After incubation, absorbance was measured at 570 nm using an iMark microplate reader (Bio-Rad, Hercules, CA, USA) with a reference wavelength of 650 nm. The cell viability (%) was determined by taking the number of viable cells without the evaluation sample as 100 when the evaluation sample was added. Each measurement was repeated twice and the average (average of values for each 12 wells) value was calculated. All results below are expressed as mean ± SE, using the Turkey method. *p* < 0.05 is considered a significant difference.

### 2.3. Confirmation of Gene Expression Involved in Hair Growth

Total RNA was extracted from HFDPCs with a mixture of WO, DPA, EPA (0.0–5.0%), and MXD (5–30 μM) in D/E solution (DMSO:Ethanol = 1:1) and incubated for 30, 60, and 90 min using the RNeasy Micro Kit (QIAGEN, Hilden, Germany). Total RNA was extracted according to the standard protocol. RNA concentration and 260/280 and 260/230 ratios were measured using a spectrophotometer to confirm RNA quality, and 100–500 ng of RNA was used to synthesize cDNA using the Affinity Script qPCR cDNA synthesis kit (Agilent, Santa Clara, CA, USA), followed by the Emerald Amp PCR Master (TAKARA, San Jose, CA, USA) with specific primers for *Fgf7* and *Vegf* genes (Table 1). PCR reactions (initial denaturation at 97 °C for 5 min, thermal denaturation at 95 °C for 45 s, annealing at 55 °C for 45 s, and extension at 72 °C for 1 min) were performed for 27 to 45 cycles. After the PCR reactions, the PCR products were separated on a 1.5% agarose gel and visualized with ethidium bromide and UV light. The expression levels of the visualized target genes were corrected for the expression level of the *Gapdh* gene, a housekeeping gene, and plotted (*n* = 3).

### 2.4. Evaluation of Hair Growth Using the Mouse Dorsal Skin Model

To evaluate the hair-growth-promoting effects of WO, a C57BL/6J mouse dorsal skin model was used for comparison. This is because MXD is used to treat alopecia [18] and is commonly used as a positive control in many hair studies. To improve the penetration of the test samples, propylene glycol: ethanol: water (5:3:2) was used as the VC group, and preliminary experiments were conducted with 1, 10, and 20% concentrations of WO. After preliminary experiments, the concentration of WO was determined to be 20%. C57BL/6J mice were divided into three groups (6 mice/group) and housed ad libitum at 22 ± 2 °C, 6:00–18:00, with a 12 h/12 h light/dark cycle. The hair cycle was adjusted by shaving the dorsal skin of the mice. Mice were shaved without anesthesia at 6 weeks of age using a trimmer MODEL 2200 (THRIVE), and 1 week later they were shaved again and the test samples were applied. Week 0 is 7 weeks old, week 1 is 8 weeks old, and week 3 is 10 weeks old. Briefly, 200 μL of the adjusted sample was applied to the back of the mice daily, and observations and photographs were taken at 0, 1, and 3 weeks. Photographs were analyzed using ImageJ software (National Institutes of Health and the Laboratory for Optical and Computational Instrumentation (LOCI, University of Wisconsin, USA)). The ratio of hair growth area to shaved area was calculated, and the hair growth rate (%) was measured.

All experimental procedures involving animals were approved by the Institutional Animal Care and Use Committee of Hoshi University. The mice were bred and maintained under specific pathogen-free conditions in the animal facility of Hoshi University.

### 2.5. Histological Observations

In the hair growth evaluation experiment, the whole dorsal skin tissue of mice was fixed with 4% paraformaldehyde solution and replaced with PBS after 3 weeks of application of the conditioned sample. Paraffin sections were dehydrated and permeated with xylene for embedding in paraffin; 5 μm paraffin sections were treated with hematoxylin/eosin stain, dehydrated, permeated, and sealed with cover glass. Section images were photographed with a BZ-X 710 brightfield system (Keyence, Kansas City, MO, USA) at ×40 magnification, and images were analyzed using ImageJ software. The total number of hair follicles in the dermis and subcutaneous tissue and the thickness of the subcutaneous tissue were measured and the mean number of hair follicles and mean thickness of the subcutaneous tissue were calculated (*n* = 6), along with the hair follicle diameter (*n* = 20).

### 2.6. Whole-Genome DNA Microarray Analysis

In hair growth evaluation experiments, whole mouse dorsal skin tissue was sampled at 1 and 3 weeks after application of the conditioned samples and stored at −80 °C. The dorsal skin tissue was pulverized to a fine powder in liquid nitrogen and stored at −80 °C until RNA isolation. Dorsal skin tissue was ground to a fine powder in liquid nitrogen and stored at −80 °C until RNA isolation. DeNovix (Wilmington, DE, USA) formaldehyde–agarose gel electrophoresis was performed. Microarray analysis was performed using the Whole Mouse Genome Oligo DNA Microarray Kit 4x44K (Agilent) and the dye-swap method [19,20] to identify genes whose expression changed after WO and MXD treatment (upregulation: UP (≥1.5-fold), downregulation: DOWN (≤0.75-fold)) compared to VC. The Functional_Categories (KEYWORDS) and pathway (KEGG pathway) of the list of variable genes selected by the microarray analysis were analyzed using Database for Annotation, Visualization and Integrated Discovery (DAVID) v6.8.

The data have been deposited with the NCBI’s Gene Expression Omnibus—GSE263884 (https://www.ncbi.nlm.nih.gov/geo/query/acc.cgi?acc=GSE263884; accessed on 13 April 2024).

## 3. Results

### 3.1. Proliferative Effects of HFDPC by MTT Assay

**Figure 1:**
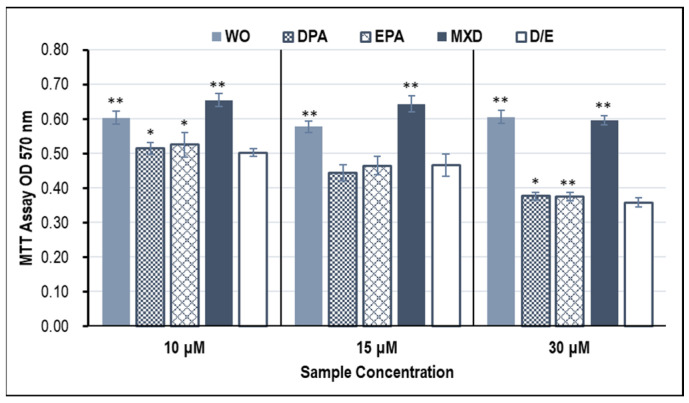
HFDPC proliferation effect of WO with the MTT assay. The graph shows the results on the third day after addition of the test sample. Results are presented as mean ± SE; ** *p* < 0.01, * *p* < 0.05 when compared to the respective D/E values by Student’s *t*-test. WO, whale oil; MXD, minoxidil; DPA, docosapentaenoic acid; EPA, eicosapentaenoic acid; D/E, DMSO:Ethanol.

The results of cell proliferation effects in the HFDPCs with MTT assay are shown in Figure 1. Significant cell proliferative effects of the whale oil (WO) and minoxidil (MXD) were observed at all concentrations of 10, 15, and 30 μM compared to the control (D/E). The proliferative effect of WO on the HFDPCs was less effective than that of WO with DPA and EPA alone, suggesting that components other than DPA and EPA were involved in the proliferative effect of WO on HFDPCs.

### 3.2. Hair Growth Marker Gene Expression Results

**Figure 2:**
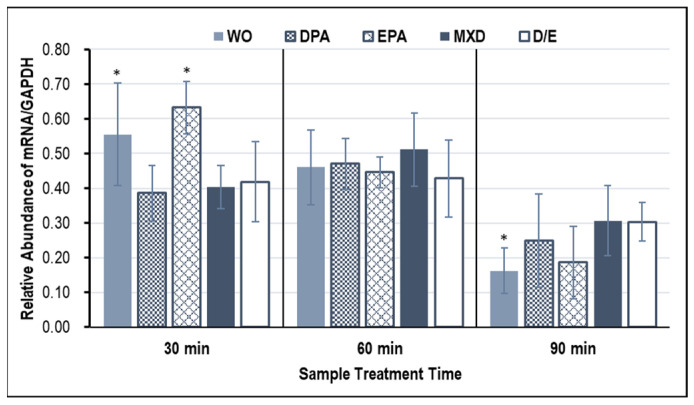
*Fgf7* gene expression in HFDPCs with RT-PCR. The graph shows the expression of the *Fgf7* gene 30, 60, and 90 min after addition of the test sample. Results are presented as mean ± SE; * *p* < 0.05 when compared to the respective D/E values by Student’s *t*-test (*n* = 3). WO, whale oil; MXD, minoxidil; DPA, docosapentaenoic acid; EPA, eicosapentaenoic acid; D/E, DMSO:Ethanol.

**Figure 3:**
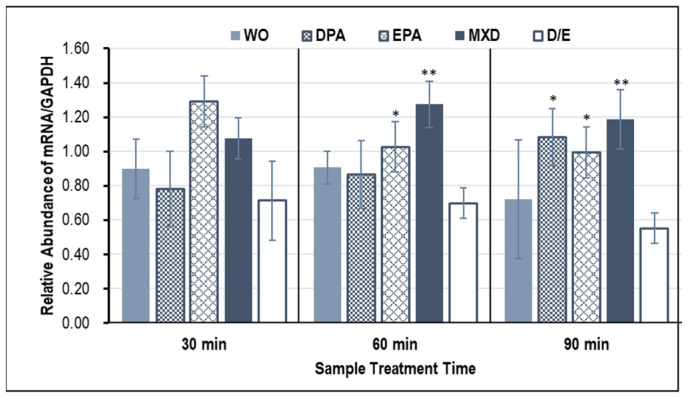
*Vegf* gene expression in HFDPCs with RT-PCR. The graph shows the expression of the *Vegf* gene 30, 60, and 90 min after addition of the test sample. Results are presented as mean ± SE; ** *p* < 0.01, * *p* < 0.05 when compared to the respective D/E values by Student’s *t*-test (*n* = 3). WO, whale oil; MXD, minoxidil; DPA, docosapentaenoic acid; EPA, eicosapentaenoic acid; D/E, DMSO:Ethanol.

Figure 2 and Figure 3 show the expression results of the hair growth marker genes, *Fgf7* and *Vegf*, in the HFDPCs induced by the WO. From the results shown in Figure 2, a significant increase in the *Fgf7* gene expression was confirmed only in WO and EPA treatment for 30 min. On the other hand, *Vegf* gene expression tended to increase at all treatment times, but significant increases in *Vegf* expression were confirmed in EPA and MXD treatments for 60 min and in DPA, EPA, and MXD treatments for 90 min (Figure 3).

### 3.3. Progression of Hair Growth

**Figure 4:**
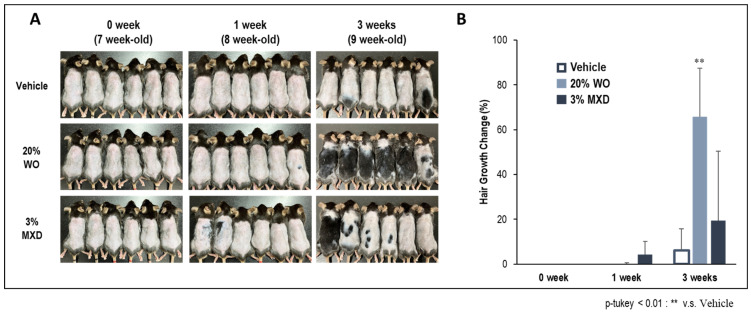
Effect of WO application on hair growth on the dorsal skin of mice. (**A**) Progressive growth of hairs on dorsal skin of mice; (**B**) percentage of hair growth area on the back of mice. WO, whale oil; MXD, minoxidil.

Figure 4A,B show the progress of hair growth in the dorsal skin of mice at 0, 1, and 3 weeks after the application of the conditioned samples. One week after the application of MXD, the hair growth rate was high, but at three weeks, the WO showed a significantly higher hair growth promotion effect. While there were individual differences in MXD, there were almost no individual differences in WO, and a high hair-growth effect was confirmed in all individuals (Figure 4A, 3 weeks).

### 3.4. Optical Microscopic Observation with H&E Images

**Figure 5:**
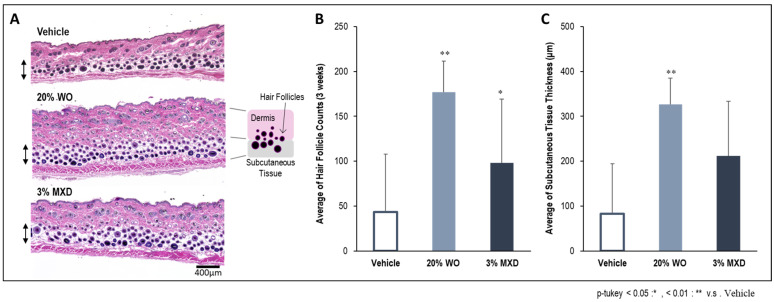
Experimental measurement of the number of hair follicles and thickness of subcutaneous tissue in the dorsal skin tissue of mice. (**A**) H&E-stained photo of mouse dorsal skin tissue; (**B**) number of hair follicles; (**C**) thickness measurement of the subcutaneous tissue. Lens magnification is ×40. WO, whale oil; MXD, minoxidil.

Figure 5 shows the results of measuring the total number of hair follicles in the dorsal skin tissue of the entire back layer of mice three weeks after application of the sample. As shown in Figure 5B, the number of hair follicles was significantly increased for both WO and MXD, but the number of hair follicles was particularly high for WO. In addition, the dorsal skin micrographs showed that the hair follicles were densely packed (Figure 5A). The thickness of the subcutaneous tissue was also measured, and a significant increase in thickness was observed for WO (Figure 5B). The thickness of subcutaneous tissue is related to: (1) protection against environmental factors and external stress and its involvement in the moisturizing function to prevent moisture loss; (2) protection of hair follicles by strengthening the formation of a layer of adipose tissue under the dermis and hair follicles, including heat insulation; and (3) indirect positive effects on hair growth by promoting the supply of necessary nutrients. Thus, it is believed that an increase in the thickness of subcutaneous tissue creates an environment conducive to healthy hair growth and further improves hair growth [21]. It was difficult to measure the length of the hair follicle, but we could measure the hair follicle diameter. As a result, a significant increase in hair follicle diameter was confirmed in WO compared to vehicle.

### 3.5. Transcriptomic Analysis Results

**Figure 6:**
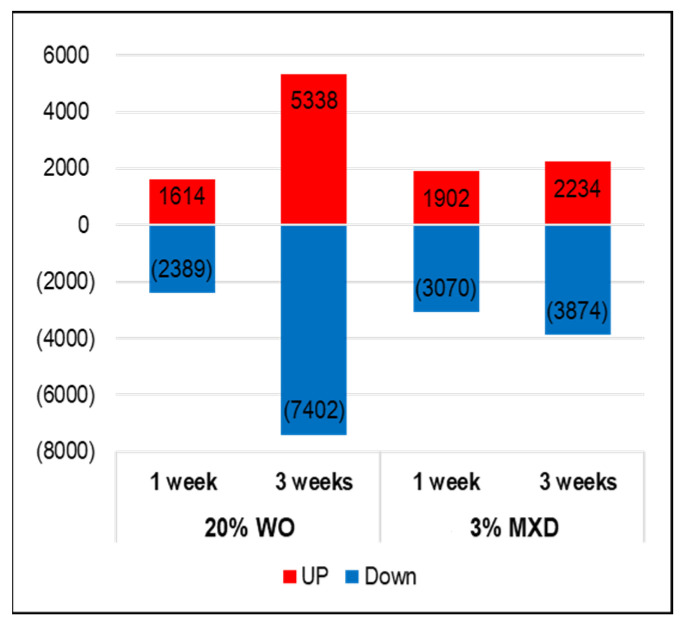
Number of genes whose expression changes were observed due to WO and MXD. The graph shows genes whose expression increased in red and the number of genes whose expression decreased in blue. WO, whale oil; MXD, minoxidil.

The cDNA microarray data have been submitted to NCBI’s Gene Expression Omnibus under the GEO series accession number GSE263884 (https://www.ncbi.nlm.nih.gov/geo/query/acc.cgi?acc=GSE263884; accessed on 13 April 2024). Figure 6 shows the results of the investigation of the expression variation gene data obtained from the DNA microarray analysis of mouse dorsal skin.

For WO, the number of genes whose expression increased (UP: red) was 1614 and the number whose expression decreased (DOWN: blue) was 2389 in the first week, and in the third week, the number of genes whose expression increased was 5338 and the number that decreased was 7402. Similarly, at week 1 MXD, there was an increase of 1902 gene transcripts and a decrease of 3070 gene transcripts, and at week 3, there was an increase of 2234 gene transcripts and a decrease of 3874 transcripts. Investigating the top 30 genes among the list of genes with variable expression identified several factors associated with hair growth. The identified genes and their brief functions are listed in Table 2. Genes colored in purple are associated with hair growth and are described below.

#### 3.5.1. Whale Oil (WO) at 1 Week

*Flg* (Filaggrin)*:* A decrease in filaggrin causes skin barrier function deterioration. Many patients with atopic dermatitis have been found to have genetic abnormalities of filaggrin, and filaggrin is attracting attention as a key substance in the treatment of atopic dermatitis. The *Flg* gene mutations also cause a decrease in natural moisturizing factors in the stratum corneum, leading to cutaneous xerosis and epithelial barrier abnormalities [22]. Filaggrin normalizes skin pH and is involved in improving barrier function [23].

#### 3.5.2. Whale Oil (WO) at 3 Weeks

*Fgf21* (Fibroblast growth factor 21)*:* It is involved in the regulation of glucose metabolism and promotes blood glucose uptake by adipocytes. Fgf21 (−/−) mice have been shown to have lower body weight, slower hair re-growth, less hair volume, and smaller hair follicle diameter compared to wild-type mice. *Fgf21* has been shown to affect hair follicle development and growth cycle in mice, suggesting that this may be related to the Pi3k/Akt and Mapk/Erk signaling pathways [24].

*Crisp1* (Cysteine-rich secretory protein 1)*:* The mouse strain C3H/HeJ, which is prone to alopecia areata, is deficient in Crisp1 protein in the hair shaft. Although clinically C3H/HeJ mice have normal hair, abnormal *Crisp1* expression may predispose them to alopecia areata and affect the severity of the disease [25].

*Padi3* (Peptidylarginine deiminase type III)*:* Padi3 is essential for hair follicle formation, and mutations in the *Padi3* gene are the cause of a rare hair disorder called uncombed hair syndrome, which is associated with centrifugal scarring alopecia of the scalp, a more frequent condition affecting mainly African women [26].

*S100a3* (S100 calcium-binding protein A3)*: S100a3* is associated with cuticle maturation and is thought to contribute to its enhancement [27]. Furthermore, blockade of S100A3 activity inhibits hair growth in mice, suggesting that *S100a3* can be used as a target for hair loss treatment [28].

#### 3.5.3. Minoxidil at 1 Week

*Shh* (Sonic hedgehog)*:* Shh is important for hair development and the hair cycle. In growing skin, *Shh* is expressed at high levels in the follicular matrix and functions as a mitogen to promote regeneration during the growth phase; SHH signaling is also essential for proper regulation of hair follicle morphogenesis [29].

*Tgm3* (Transglutaminase 3)*:* Tgm3 is involved in various processes related to hair follicle morphogenesis, promoting hair formation and strengthening and also supporting hair shaft growth. Many Tgm3 −/− mice exhibit hair abnormalities and have been found to have distorted cuticles. In addition, *Tgm3* contributes to the maintenance of skin barrier integrity [30].

#### 3.5.4. Minoxidil at 3 Weeks

*Degs2* (sphingolipid delta (4)-desaturase 2): Degs2 is a ceramide synthase, and reduced ceramide levels and altered ceramide composition are associated with atopic dermatitis and psoriasis. It has been suggested to be important for skin barrier function [31].

### 3.6. Biological Functional Enrichment Analysis

**Figure 7:**
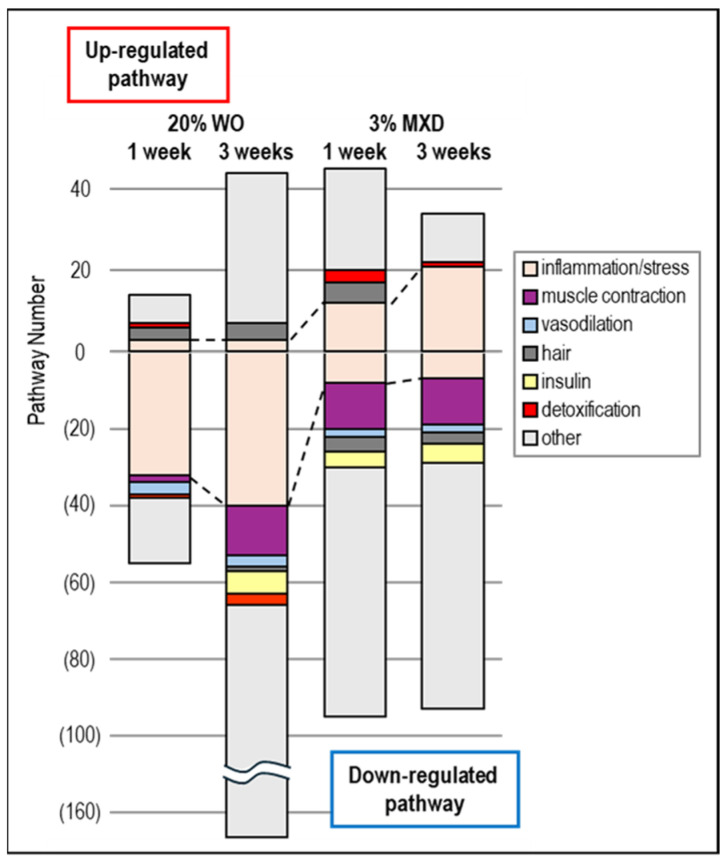
Pathway analysis of the genes with altered expression in the mouse dorsal skin. Data obtained from KEGG pathway analysis using the DAVID tool were categorized into inflammation, muscle contraction, vasodilation, hair, insulin, detoxification, and others, and the number of pathways were counted and graphed. WO, whale oil; MXD, minoxidil.

To ascertain what biological function pathways were associated with the variable expression gene clusters obtained by DNA microarray analysis of the WO treatment, we performed an enrichment analysis using the DAVID tool. Each pathway in the resulting KEGG pathway was statistically significant (*p*-value ≤ 0.05), including detoxification (mmu00480:Glutathione metabolism, mmu00983:Drug metabolism—other enzymes), insulin (mmu04911:Insulin secretion, mmu04910:Insulin signaling pathway, etc.), hair (mmu04916:Melanogenesism, mmu04310:Wnt signaling pathway [32], etc.), inflammation and stress response (mmu04060:Cytokine-cytokine receptor interaction, mmu04062:Chemokine signaling pathway, etc.), muscle contraction (mmu04270:Vascular smooth muscle contraction, mmu04260:Cardiac muscle contraction, mmu04924:Renin secretion, etc.), and vasodilation (mmu04022:cGMP-PKG signaling pathway, mmu04926:Relaxin signaling pathway, etc.), and is categorized and shown graphically in Figure 7.

**Figure 8:**
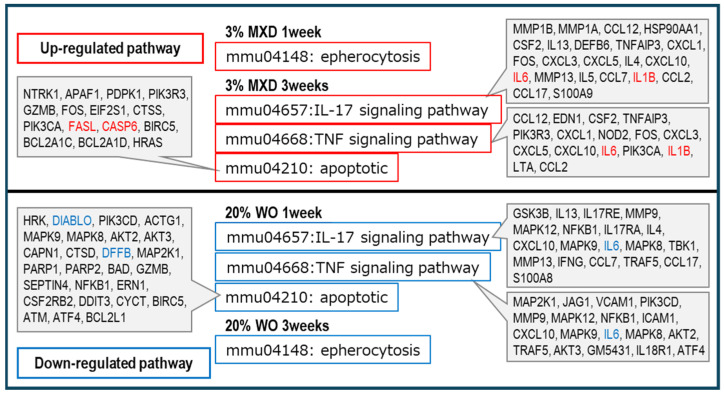
Discussion of inflammation-related and apoptosis pathway analysis results. The pathway terms selected to contrast MXD and WO in the KEGG pathway analysis classified as inflammation were mmu046657: IL-17 signaling pathway, mmu04668: TNF signaling pathway, mmu04210: apoptotic, and mmu04148: epherocytosis. WO, whale oil; MXD, minoxidil.

As shown in Figure 7, the vasodilation and muscle contraction pathways were common in the DOWN category, but the percentage of the muscle contraction pathway was particularly high in the DOWN category for both WO and MXD. This was thought to be most likely due to the promotion of vasodilation by decreasing vasoconstriction-related gene expression. These results suggest that MXD has a vasodilating effect on blood flow by dilating blood vessels, and WO may have a similar vasodilating effect. In addition, inflammation-related pathways were observed in both the UP and DOWN categories, but MXD was found to have a higher proportion of inflammation pathways in the UP category. Further confirmation of this inflammatory pathway revealed that the mmu04657:IL-17 signaling pathway and mmu04668:TNF signaling pathway, which are known to play a central role as regulators of inflammatory responses, were upregulated in the MXD signaling pathway and are known to be upregulated by MXD, whereas they were downregulated by WO. Furthermore, the apoptosis and epherocytosis pathways were also classified as upregulated in MXD and downregulated in WO. The results are summarized in Figure 8. More interestingly, insulin-related pathways were found to be predominantly DOWN for all WO and MXD categories (Figure 7).

## 4. Discussion

Many factors related to hair growth were detected by the DNA microarray analysis. As shown in Table 2, many of the upregulated genes were found to be related to anti-inflammation, hair growth, and skin barrier for WO and to hair growth, skin barrier, and melanin synthesis for minoxidil (MXD). In addition, many of the downregulated genes were found to be related to “vasoconstriction” and “muscle contraction”. Furthermore, KEGG pathway analysis (Figure 7) confirmed that pathways classified as muscle contraction-related were downregulated. This suggests that WO and MXD may cause muscle relaxation and vasodilation as a common function. Minoxidil was developed by an American pharmaceutical company in the 1960s as an antihypertensive for hypertension due to its vasodilating effect. It is believed to have a hair-growth effect from the improvement in blood flow due to this vasodilatation [33,34]. The present results suggest that WO has the same vasodilator effect as MXD.

Furthermore, as to the point that the insulin-related pathway was DOWN for both WO and MXD, diazoxide, the only drug approved by the U.S. Food and Drug Administration for the treatment of hyperinsulinemic hypoglycemia, has been shown to increase blood glucose by inhibiting insulin secretion through opening of ATP-sensitive potassium (KATP) channels. Hypertrichosis has been reported as a side effect [35,36], and indeed diazoxide is also known to stimulate hair growth [37]. Like diazoxide, MXD is a potent KATP channel opener, and as a result, decreased expression of many genes classified in the insulin secretion-related pathway were detected. Whale oil also showed a large decrease in the expression of genes classified in the insulin secretion-related pathway, suggesting that WO may also have a KATP-channel-opening effect.

IL17 and TNF signals are known to play a central role as regulators of inflammatory responses. As shown in Figure 8, IL17 and TNF signals, which promote inflammation, were increased by MXD and decreased by WO. IL-1B, which was included in the IL17 and TNF signals in MXD, is a potent proinflammatory cytokine that is rarely detected in normal tissues and is produced and secreted by inflammation-inducing stimuli [38].

In addition, for mmu04210: apoptotic and mmu04148: epherocytosis, MXD was classified as an ‘increase’, and increased expression of FASL and CASP6, which are involved in activating apoptosis, was confirmed. On the other hand, WO was classified as a ‘decrease’, and decreased expression of DIABL and DFFB, which activate apoptosis, was observed (Figure 8).

Apoptosis is promoted by intracellular stresses such as oxidative stress, and furthermore, epherocytosis has an inflammation-reducing effect that processes dead cells before toxic reactive oxygen species, proteases, and caspases are released by a mechanism in which dead cells are removed by phagocytes after apoptosis [39]. It has also been suggested that reducing apoptosis promotes hair growth and minimizes hair loss [40]. Therefore, considering these results, it is possible that the WO may promote hair growth more than MXD by inhibiting or reducing apoptosis.

The side effects of MXD include dermatitis such as eczema, hives, and itching, and the probability of MXD-induced side effects is estimated to be about 8%. The propylene glycol contained in the solvent used to penetrate MXD may be considered as a cause of the high incidence of dermatitis, but propylene glycol itself is not the cause because the solvent used for WO in this experiment also contained propylene glycol. Therefore, the results of this current research suggest that although dermatitis symptoms were not observed, inflammation and oxidative stress may have increased with MXD compared to WO at the transcript level. These results suggest that WO, like MXD, is a vasodilator and may promote hair growth by reducing inflammation, oxidative stress, and apoptosis.

## 5. Conclusions

This study on the effect of WO using genomics data and histological experiments provided insight into transcript-wide changes and highlighted some potential mechanisms and factors involved in hair growth. The study is not exhaustive and needs further research, and there are certain limitations to this study, as follows: (1) There were no terms related to hair growth that were UP or DOWN only for the WO. However, there are changes (oppositely regulated) in gene expression among WO and MXD. For example, we did identify that the Wnt signaling pathway, which is said to be related to hair growth, was UP in the third week of WO and DOWN in the third week of MXD. Since this experiment evaluated hair growth in HFDPCs and mice, the actual hair growth effect of WO in humans needs to be confirmed in future experiments/human trials. Furthermore, the hair growth mechanism of WO was analyzed only at 0, 1, and 3 weeks by whole-genome DNA microarray expression analysis in this experiment. Therefore, it is necessary to elucidate the mechanism in detail, including the application of RT-PCR and Western blotting approaches for quantification of the target genes and protein components, taking the hair cycle into consideration. (2) It is also necessary to confirm the proliferative effect of not only hair papilla cells but also the hair follicle stem cells (HFSCs). Since the number of HFSCs was not measured in this experiment, it is not clear whether the action of WO is to increase or activate HFSCs. However, the function of the SHH signaling pathway promotes activation and proliferation of quiescent HFSCs (the fold-change of the *Shh* gene was found to be 2.08 at 1 week of WO and 3.56 at 3 weeks) [41]. Furthermore, HFSCs proliferate by receiving hair-growth-promoting signals from the papilla cells [42], and FGF-7 and VEGF have been shown to be important factors for the proliferation of HFSCs [43]. Based on these facts, it is conceivable that WO may proliferate HFSCs via papilla cells. Moreover, ki67 and Edu immunofluorescence staining methods would be more accurate for measuring the proliferation of the HFSCs in mouse dorsal skin.

## Figures and Tables

**Table 1:** Primer designs used for RT-PCR analysis.

	Forward Primer		Reverse Primer			
Accession (Gene)	Primer Name	Nucleotide Sequence (5′-3′)	Primer Name	Nucleotide Sequence (5′-3′)	Product Size (bp)	Gene Name
NM_002009	hH005	cacagataggaagaggtcaatga	hH006	taacttcttgtgtgtcgctcag	262	*Fgf7*
AY047581	hH007	ctgaggagtccaacatcaccat	hH008	ttcgtttaactcaagctgcctc	254	*Vegf*
NM_002046	hH009	acagtcagccgcatcttctt	hH010	atgggatttccattgatgaca	276	*Gapdh*

**Table 2:** Factors related to hair growth in the top genes of expression variation. (in red: increased expression; in blue: decreased expression).

**20% WO 1 week**			**20% WO 3 weeks**		
Gene name	Fold	Function	Gene name	Fold	Function
*Mug2*	12.94	Anti-inflammatory, Anti-degranulation	*Fgf21*	7.39	Hair Growth, Barrier Function
*Scgb1b27*	7.37	Anti-inflammatory	*Crisp1*	7.42	Alopecia
*Scgb1b26-ps*	6.76	Anti-inflammatory	*Idi2*	7.79	Moisturizing
*Mug1*	5.82	Anti-inflammatory, anti-degranulation	*Serpina1d*	7.13	Anti-inflammatory
*Flg*	5.26	Anti-inflammatory, Moisturizing, Barrier function	*S100a7a*	6.27	Skin Protection
*Idi2*	4.82	Moisturizing	*Tchh*	4.71	Moisturizing, Barrier function
*Stfa1*	4.73	Anticoagulant	*Padi3*	4.52	Hair Growth, Barrier Function
*Scgb1b30*	4.72	Anti-inflammatory	*S100a3*	4.00	Cuticle
*Slc16a6 (MCT7)*	0.18	Inflammation	*Pon1*	0.08	Antioxidant
*Cndp2*	0.20	Oxidation	*Myh3*	0.11	Muscle contraction
*Diablo*	0.23	Apoptosis	*Sln*	0.11	Muscle contraction
*Acod1*	0.27	Oxidation/Inflammation	*Pvalb*	0.13	Muscle contraction
*Pyy*	0.27	Vasodilation	*Mylpf*	0.13	Muscle contraction
*Slc5a3*	0.28	Oxidation/Inflammation	*Neb*	0.14	Muscle contraction
*Renbp*	0.29	Vasoconstriction	*Ttn*	0.14	Muscle contraction
*Slc5a3 (SMIT1)*	0.31	Vasoconstriction	*Ptgs1(COX-1)*	0.14	Vasoconstriction
**3% MXD 1 week**			**3% MXD 3 weeks**		
Gene name	Fold	Function	Gene name	Fold	Function
*Oca2*	54.35	Melanin synthesis	*Sprr2i*	10.10	Barrier function
*Calhm4*	45.11	Barrier function	*Mug2*	9.32	Anti-inflammatory, Anti-degranulation
*S100a7a*	33.73	Skin Protection	*Sprr2a3*	6.94	Barrier function
*Trpm1*	34.02	Melanin synthesis	*Sprr2j-ps*	5.73	Barrier function
*Padi3*	30.89	Hair Growth, Barrier Function	*Tff1*	4.76	Barrier function
*Shh*	28.83	Hair growth	*Nppb*	4.71	Vasorelaxation
*S100a3*	15.34	Cuticle	*Sprr2e*	4.39	Barrier function
*Tgm3*	4.68	Hair Growth, Barrier Function	*Degs2*	4.35	Barrier function
*Leap2*	0.24	Antibacterial	*Mgst3*	0.08	Detoxification
*Serpinb12*	0.26	Skin Protection	*Slc25a15*	0.14	Detoxification
*Hamp2*	0.27	Antimicrobial	*Slc22a4*	0.14	Detoxification
*Myh4*	0.31	Muscle contraction	*Ptx3*	0.19	Anti-inflammatory
*Ttn*	0.33	Muscle contraction	*Prdx3*	0.21	Antioxidant
*Myh8*	0.33	Muscle contraction	*Mylpf*	0.23	Muscle contraction
*Myh2*	0.35	Muscle contraction	*Myh8*	0.24	Muscle contraction
*Myh1*	0.40	Muscle contraction	*Des*	0.26	Muscle contraction

## Data Availability

The data presented in this study are available in the article and submitted databases. The raw data are available upon reasonable request from the corresponding author.
